# Loss of FOXC1 contributes to the corneal epithelial fate switch and pathogenesis

**DOI:** 10.1038/s41392-020-00378-2

**Published:** 2021-01-08

**Authors:** Mingsen Li, Liqiong Zhu, Jiafeng Liu, Huaxing Huang, Huizhen Guo, Li Wang, Lingyu Li, Sijie Gu, Jieying Tan, Jing Zhong, Bowen Wang, Zhen Mao, Yong Fan, Chunqiao Liu, Jin Yuan, Hong Ouyang

**Affiliations:** 1 State Key Laboratory of Ophthalmology, Zhongshan Ophthalmic Center, Sun Yat-sen University, 510060 Guangzhou, China; 2 Key Laboratory for Major Obstetric Diseases of Guangdong Province, The Third Affiliated Hospital of Guangzhou Medical University, 510150 Guangzhou, China

**Keywords:** Adult stem cells, Cell biology

## Abstract

Forkhead box C1 (FOXC1) is required for neural crest and ocular development, and mutations in *FOXC1* lead to inherited Axenfeld–Rieger syndrome. Here, we find that FOXC1 and paired box 6 (PAX6) are co-expressed in the human limbus and central corneal epithelium. Deficiency of FOXC1 and alternation in epithelial features occur in patients with corneal ulcers. FOXC1 governs the fate of the corneal epithelium by directly binding to lineage-specific open promoters or enhancers marked by H3K4me2. *FOXC1* depletion not only activates the keratinization pathway and reprograms corneal epithelial cells into skin-like epithelial cells, but also disrupts the collagen metabolic process and interferon signaling pathways. Loss of interferon regulatory factor 1 and PAX6 induced by FOXC1 dysfunction is linked to the corneal ulcer. Collectively, our results reveal a FOXC1-mediated regulatory network responsible for corneal epithelial homeostasis and provide a potential therapeutic target for corneal ulcer.

## Introduction

Corneal integrity and transparency are essential for normal vision. The outermost surface of the cornea is covered with a non-keratinized stratified squamous epithelium consisting of multilayer corneal epithelial cells (CECs) on the avascular stroma. The self-renewal and regeneration of the corneal epithelium are sustained by a population of limbal stem cells (LSCs), which are segregated in the basal layer of the limbal zone of the peripheral cornea.^1,2^ LSCs continuously generate committed transient amplifying cells that migrate toward the central cornea and differentiate into mature CECs.^3,4^ However, various insults including chronic inflammation, infection, Stevens–Johnson syndrome, and genetic mutations lead to LSC dysfunction, causing corneal blindness characterized by opacification, neovascularization, and conjunctivalization of the cornea.^5,6^

Cell fate determination and identity are governed by lineage-specific transcription factors (TFs) and epigenetic modifications. Key TFs not only determine organ development, but also define tissue specificity during adulthood. The master regulator paired box 6 (PAX6) plays a vital role in the developing visual system, and is required for corneal epithelial identity and homeostasis.^7–9^ Loss of PAX6 induces the corneal epithelium to acquire an opaque skin-epithelium phenotype, which leads to visual disorder.^8^

Forkhead Box C1 (FOXC1) is a key TF of ocular development.^10^ *FOXC1* mutations in human patients cause ocular anterior segment defects, and pathological corneal neovascularization can be observed in patients with Axenfeld–Rieger syndrome harboring *FOXC1* mutations or copy-number alterations.^10,11^ Previous findings indicated that Foxc1 maintains corneal transparency and avascularity in the neural crest-derived corneal stromal and endothelial cells.^11^ Neural crest-specific *Foxc1* null mutation in mice can result in disorganized corneal stroma with excessive growth of blood vessels and failure of corneal endothelial formation.^11^ However, the role of FOXC1 in the corneal epithelial homeostasis and pathogenesis has not been reported.

In the present study, we identify FOXC1 as a novel core regulator that underlies the lineage commitment of human corneal epithelium. FOXC1 regulates the corneal epithelial lineage-specific gene expression and interferon signaling pathways by occupying open *cis*-regulatory elements that are marked by histone H3 lysine 4 dimethylation (H3K4me2). *FOXC1*-depleted LSCs show the activation of an epidermis-specific gene program and the repression of IRF1 and PAX6, which are pathological characteristics of the human corneal ulcer. These findings indicate that the FOXC1 regulatory network is required for corneal epithelial homeostasis, and FOXC1 may serve as a potential target for corneal ulcer treatment.

## Results

### Co-expression of FOXC1 and PAX6 in the corneal epithelium

**Fig. 1:**
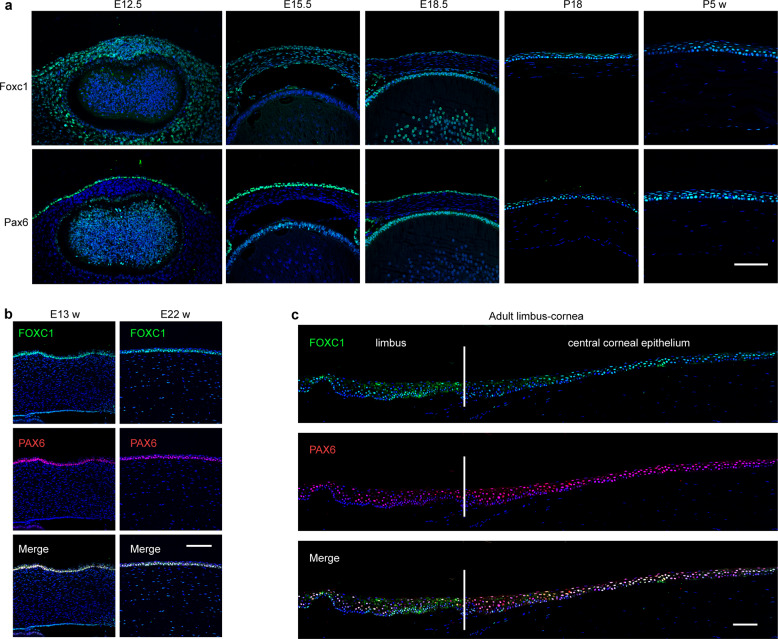
FOXC1 and PAX6 are co-expressed in the limbus and central cornea during development. **a** Immunofluorescence staining of Foxc1 and Pax6 during mouse corneal development (E12.5, E15.5, E18.5, postnatal day 18.5, and postnatal 5 weeks). Scale bars, 100 μm. Blue = DAPI, Green = Foxc1/Pax6. **b** Immunofluorescence staining of FOXC1 and PAX6 in the human cornea at E13 and E22 weeks. Scale bars, 100 μm. **c** Immunofluorescence staining of FOXC1 and PAX6 in the limbus and corneal epithelium of adult human. Scale bar, 100 μm

To determine whether FOXC1 functions in the corneal epithelium, we first detected its expression pattern during corneal development. We found that Foxc1 was evidently expressed in the ocular surface structure and neural crest-derived cells during the early developmental stage in mice (Fig. 1a). The corneal epithelium showed Foxc1 expression from the embryonic developmental stage to adulthood in mice (Fig. 1a). Foxc1 was detected in the corneal stroma at embryonic day 15.5 (E15.5). In contrast, Pax6 was expressed in the outmost layer of the mouse ocular surface at E12.5. Pax6 was also expressed in the corneal epithelium but not in the corneal stroma during development and adulthood (Fig. 1a).

As expected, FOXC1 and PAX6 co-expressed robustly in the human corneal epithelium of 13-week and 22-week-old embryos (Fig. 1b). Their expressions were also maintained in the adult limbus and central corneal epithelium (Fig. 1c). These observations implied that FOXC1 may play an important role in corneal epithelial development.

### Loss of FOXC1 and PAX6 is associated with human corneal ulcer

**Fig. 2:**
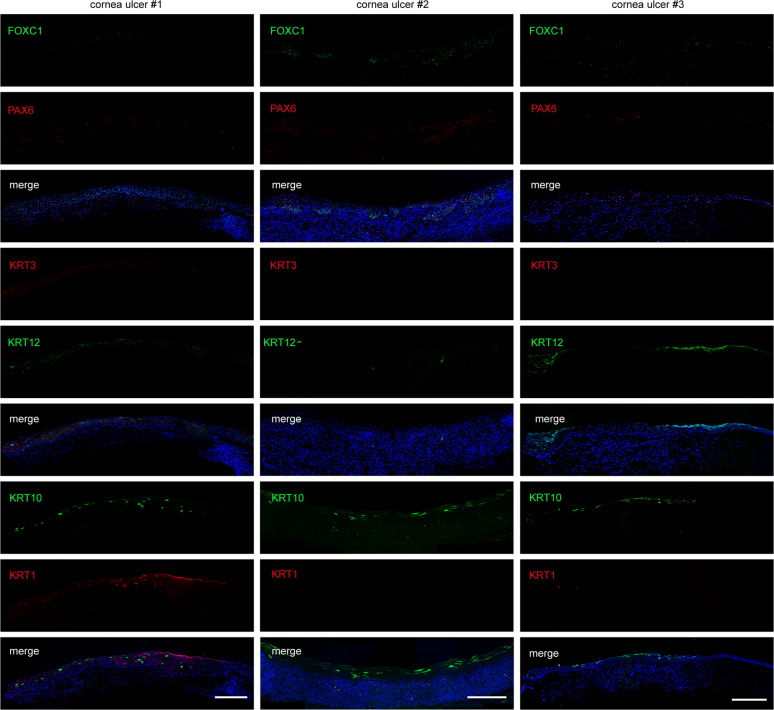
Aberrant expression of FOXC1 and PAX6 in the human corneal ulcer tissues. Immunofluorescence staining of FOXC1, PAX6, KRT3, KRT12, KRT1, and KRT10 in the human corneal ulcer tissues. Scale bar, 100 μm

To study the potential relationship between FOXC1 and corneal pathogenesis, we examined 21 biopsy samples from patients with corneal ulcer. Remarkably, partial regions of the diseased corneal tissues from nine patients showed conspicuous absence of FOXC1, PAX6, and the corneal epithelial markers (KRT3 and KRT12). Instead, the keratinized epithelium-specific KRT1 or KRT10 appeared in these lesion areas (Fig. 2). These observations demonstrated the disruption of epithelial identity and homeostasis in corneal ulcer, and suggested that FOXC1 deficiency was probably associated with disorder of the corneal epithelium.

### Gene expression program of CEC differentiation identifies FOXC1 as a key regulator

**Fig. 3:**
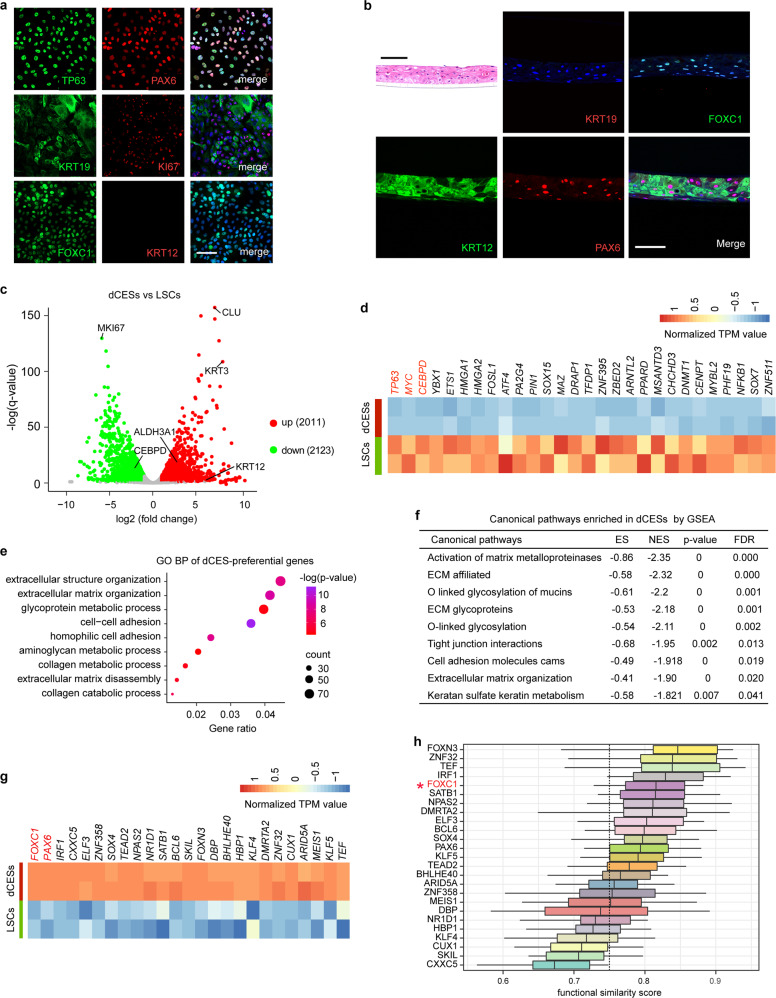
Gene network of the corneal epithelial differentiation. **a** Immunofluorescence staining of the indicated genes in primary LSCs. Scale bars, 50 μm. **b** Hematoxylin and eosin staining (upper left) in the dCESs induced through air-lifting culture. Immunofluorescence staining of the indicated genes in the dCESs induced through air-lifting culture. Scale bar, 100 μm. **c** Volcano plot showing gene expression levels in LSCs versus those in the dCESs. **d** Heatmap showing normalized transcripts-per-million reads (TPM) values of the downregulated TFs in the dCESs. **e** GO biological process (BP) analysis of genes expressed at higher levels in the dCESs than in LSCs. **f** GSEA showing the canonical pathways enriched in the preferentially expressed gene set in the dCESs. **g** Heatmap showing normalized TPM values of the top 25 (fold change) upregulated TFs with high expression levels in the dCESs. **h** GSSM of the top 25 (fold change) upregulated TFs with high expression levels in the dCESs. The cut-off value (0.75) is indicated by the dashed line

We then amplified human LSCs ex vivo and identified them by LSC markers KRT19, TP63, and PAX6 (Fig. 3a).^12,13^ Our primary LSCs showed a high proliferative potential, as evidenced by robust expression of cell proliferative marker KI67 (Fig. 3a). An air-lifting culture system was previously developed as a superior three-dimensional (3D) differentiation model of stratified epithelia.^14,15^ In this 3D model, LSCs were seeded into inserts of transwell plates and expanded in the culture medium. After the LSCs reached confluency, the medium in the upper chambers was removed to expose the cells to the air, and a small volume of medium in the lower chamber contacted with the cells to provide nutrition, which mirrors the condition under which CECs differentiate in vivo. Following induction in an air-lifting culture system, we obtained multilayer differentiated corneal epithelial sheets (dCESs) (Fig. 3b). Expression of PAX6 and CEC marker KRT12^4,16^ (but not KRT19) in the dCESs indicated their terminal differentiation (Fig. 3b). In parallel with the in vivo-expression pattern, FOXC1 was also robustly expressed both in LSCs and in dCESs (Fig. 3a, b).

To explore the gene regulation program of corneal epithelial differentiation, we performed differential gene expression analysis for RNA-Seq data between LSCs and dCESs. The dCESs withdrew from cell-cycle progression, as evidenced by loss of *KI67* (Fig. 3c). In addition, we obtained a set of TFs that were downregulated upon differentiation, including *TP63*, *MYC*, and *CEBPD*,^17^ which were well demonstrated to promote LSC proliferation (Fig. 3d).

In contrast, we identified 2011 genes that were associated with CEC differentiation, such as the corneal epithelial markers *KRT3*,^18^ *KRT12*, *ALDH3A1*,^19^ and *CLU*^20^ (Fig. 3c). Gene ontology (GO) analysis showed that genes preferentially expressed in dCESs are linked to several biological processes, such as extracellular structure/matrix organization, cell–cell adhesion, glycoprotein metabolic process, and collagen metabolic process (Fig. 3e). Gene set enrichment analysis (GSEA) revealed that multiple canonical pathways, including activation of matrix metalloproteinases, O-linked glycosylation of mucins, extracellular matrix glycoproteins, tight junction interactions, cell adhesion molecules cams, extracellular matrix organization, and keratan sulfate keratin metabolism, were significantly upregulated during CEC differentiation (Fig. 3f).

We also identified a cohort of TFs that were highly expressed and upregulated in dCESs, including *FOXC1*, *PAX6*, and the well-known pro-differentiation regulators *KLF4* and *KLF5*^21^ (Fig. 3g). The parallel expression pattern observed for FOXC1 and PAX6, both ex vivo and in vivo, implied that FOXC1 may play a key role in the corneal epithelium. In addition, to identify the core regulators of CEC differentiation, we selected the top 25 upregulated TFs by fold changes and computed their functional similarities by GO-terms Semantic Similarity Measures (GSSM, Fig. 3h), a tool for semantic comparisons of GO annotations.^22^ The high functional similarity score of FOXC1 implied that it was a hub regulator of these 25 candidates.

### FOXC1 occupies the H3K4me2-marked *cis*-regulatory elements

In general, TFs selectively bind to enhancers or promoters to control transcriptional programs. We mapped the genome-wide binding profile of FOXC1 in LSCs using chromatin immunoprecipitation sequencing (ChIP-Seq). ChIP-Seq data of H3K4me2, which is associated with both the transcription start sites (TSSs) and enhancers,^23,24^ was used to delineate the epigenetic feature of LSCs. The accessible chromatin was assessed by assay for transposase-accessible chromatin with sequencing (ATAC-Seq).^25^ These high-throughput sequencing data showed a high Pearson’s correlation coefficient between two biological replicates (Supplementary Fig. S1).

**Fig. 4:**
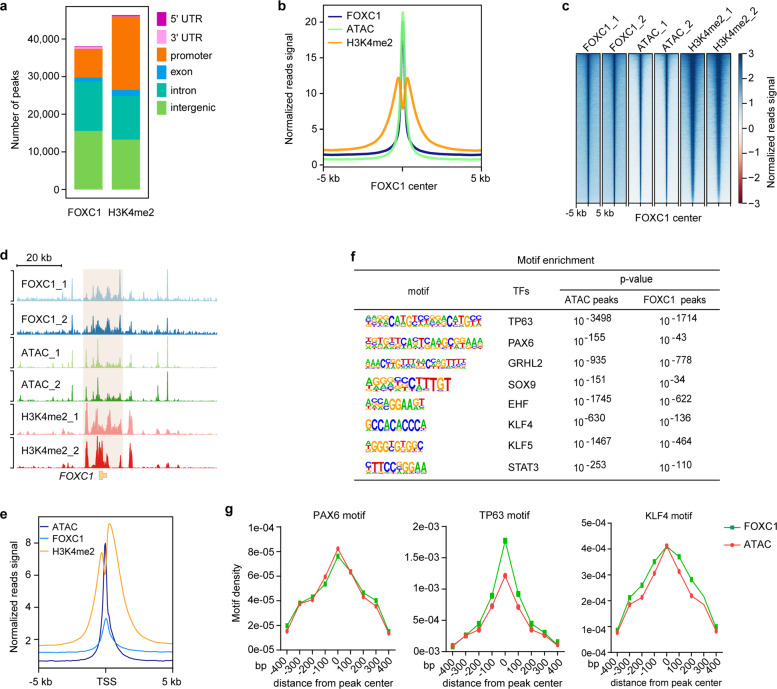
Chromatin profiles of FOXC1 occupancies in LSCs. **a** Barplot showing the distribution patterns of FOXC1 and ATAC peaks in LSCs. **b** Metaplot showing the average FOXC1, H3K4me2, and ATAC enrichment signals around the center of FOXC1 peaks. **c** Heatmaps showing the binding signals of FOXC1, ATAC, and H3K4me2 at the center of FOXC1 peaks. **d** Genome browser tracks for FOXC1, H3K4me2, and ATAC enrichment signals across the *FOXC1* locus. **e** Metaplot showing the average FOXC1, H3K4me2, and ATAC enrichment signals around the center of TSSs. **f** Motifs enriched in ATAC and FOXC1 peaks. **g** Frequencies of PAX6, TP63, and KLF4 motifs around the centers of FOXC1 and ATAC peaks

We found that FOXC1 principally bound to intronic and intergenic regions, and a small portion (~20%) of FOXC1 peaks was located at proximal promoters. In contrast, ~42% of H3K4me2 peaks were located at promoters and ~50% marked putative noncoding enhancers (Fig. 4a). ATAC and H3K4me2 signals were significantly enriched at the center of FOXC1 peaks (Fig. 4b). Genome-wide binding heatmaps showed that FOXC1 colocated with ATAC and H3K4me2 (Fig. 4c), suggesting that FOXC1 occupied open *cis*-regulatory elements. Interestingly, FOXC1 bound to its own promoter and multiple neighboring enhancers that were marked by H3K4me2 and ATAC (Fig. 4d), forming an autoregulatory loop. In addition, FOXC1, ATAC, and H3K4me2 were also significantly enriched at the TSSs (Fig. 4e).

Accessible chromatin regulatory elements can recruit TFs to control gene transcription programs. The function and identity of cells or tissues are generally dominated by cooperation between multiple lineage-specific TFs. Thus, we used the HOMER algorithm^26^ to search TF binding motifs in the FOXC1 and ATAC peaks. We found that both ATAC and FOXC1-bound regions were significantly coenriched for the motifs of key corneal epithelial regulators (TP63, PAX6, GRHL2,^27,28^ SOX9,^29^ EHF,^30^ KLF4, KLF5, and STAT3;^31^ Fig. 4f). These motifs were located at the center of the FOXC1 and ATAC peaks (Fig. 4g and Supplementary Fig. S2). Collectively, we delineated the chromatin landscapes of FOXC1 binding sites.

### FOXC1 determines the corneal epithelial fate via activating PAX6 and repressing epidermal regulators

To explore the function and molecular mechanism of FOXC1 in the corneal epithelium, we performed short-hairpin RNA (shRNA)-mediated knockdown experiments. ZNF750 is known to initiate early- and late-differentiation programs in skin epithelial cells.^32^ We found that *FOXC1* depletion significantly inhibited *PAX6* expression and activated *ZNF750* in LSCs (Supplementary Fig. S3a and S3b), suggesting that a cellular identity switch was triggered. About a third (107/194) of the downregulated genes in shFOXC1-treated LSCs were preferentially expressed in dCESs (Supplementary Fig. S3c), suggesting that FOXC1 plays a role in corneal epithelial differentiation. However, *PAX6* depletion did not significantly affect expression of *FOXC1* in LSCs (Fig. S3d). These results indicated that FOXC1 is the upstream regulator of PAX6. Thus, we speculated that FOXC1 dysfunction altered the lineage commitment of LSCs.

**Fig. 5:**
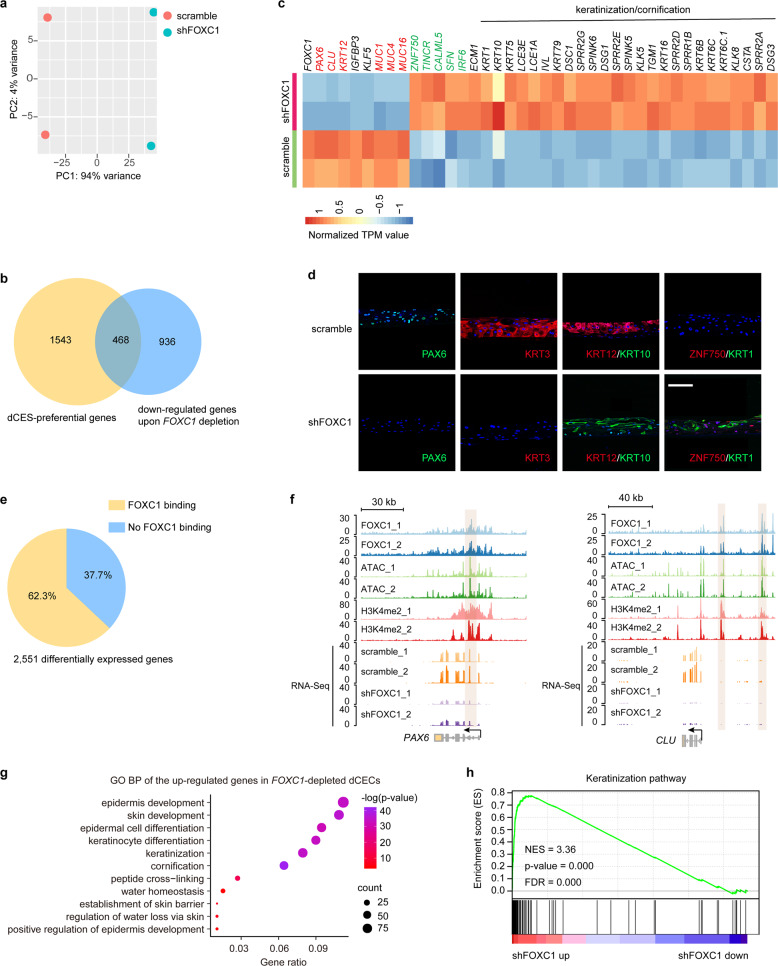
*FOXC1* depletion induces differentiation of LSCs into skin-like keratinized epithelium. **a** PCA of the air-lifting-induced dCESs treated with scrambled shRNA and shFOXC1. **b** Venn diagram showing the overlap between genes preferentially expressed in the dCESs and those that were downregulated after *FOXC1* knockdown in the dCESs. **c** Heatmap showing normalized TPM values of the selected differentially expressed genes in scrambled shRNA-treated and shFOXC1-treated dCESs. **d** Immunofluorescence staining of the indicated genes in scrambled shRNA- and shFOXC1-treated dCESs. Scale bar, 100 μm. **e** Pie chart showing the percentages of differentially expressed genes, with or without FOXC1 binding, that were induced by *FOXC1* knockdown in the dCESs. **f** Genome browser tracks for FOXC1, H3K4me2, and ATAC enrichment signals across the *PAX6* and *CLU* loci in LSCs. **g** GO BP analysis of genes upregulated in the *FOXC1*-depleted dCESs. **h** GSEA of keratinization pathway in the gene expression matrix of scrambled shRNA- versus shFOXC1-treated dCESs

Next, *FOXC1*-deficient LSCs were induced to differentiate into stratified epithelium using the 3D air-lifting culture system. Although epithelial stratification was not affected when *FOXC1* was knocked down, the morphology and arrangement of the dCESs were disordered (Fig. S4a). RNA-Seq analysis was used to determine the FOXC1-regulated gene network during differentiation. Hierarchical clustering showed a high degree of agreement between the two biological replicates (Supplementary Fig. S4b). Principal component analysis (PCA) indicated that gene expression of *FOXC1*-depleted dCESs was quite distinct from that of the control group (Fig. 5a). We identified 1404 downregulated genes and 1148 upregulated genes in shFOXC1-treated dCESs (Supplementary Fig. S4c).

*FOXC1* knockdown in dCESs downregulated 468 differentiation-associated genes (Fig. 5b), including the corneal epithelium marker genes *PAX6*, *KRT3*, *KRT12*, *CLU*, *MUC1*, *MUC4*, and *MUC16*^33^ (Fig. 5c, d and Supplementary Fig. S4d). GSEA also revealed an overall compromise of the differentiation-associated gene program in *FOXC1*-depleted dCESs (Supplementary Fig. S4e). Of note, *FOXC1* inhibition only induced 745 differentially expressed genes in LSCs, which was far less than that (2551) in dCESs. A total of 2180 genes were regulated by FOXC1 only during CEC differentiation (Supplementary Fig. S4f). These results suggested that FOXC1 plays a vital role in the CEC differentiation.

The chromatin binding profile showed that a large percentage (62.3%) of the differentially expressed genes induced by *FOXC1* knockdown was directly bound by FOXC1 (Fig. 5e). For example, FOXC1 occupied ATAC-marked and H3K4me2-marked enhancer elements of its downstream genes *PAX6*, *CLU*, *ST3GAL4*, and *WNT7B* (Fig. 5f and Supplementary Fig. S5a). FOXC1 also bound to the activated promoters of *WNT7B*, *ST3GAL4*, *MUC1*, and *IGFBP3*, which were repressed upon *FOXC1* depletion (Supplementary Fig. S5a, b).

Furthermore, we observed activation of the epidermis-specific genes *ZNF750*, *TINCR*, *CALML5*, *SFN*, *IRF6*, *KRT1*, and *KRT10*^8^ when *FOXC1* was knocked down (Fig. 5c, d and Supplementary Fig. S4d). ZNF750 and TINCR are known to promote epidermal differentiation by inducing CALML5, which interacts with SFN.^34^ IRF6 is required for skin epithelial differentiation and barrier function.^35,36^ GO analysis showed that *FOXC1*-deficient dCESs possessed epidermis function and identity, evidenced by the upregulation of the gene program controlling skin epithelium-specific biological processes, like epidermal cell differentiation, keratinization/cornification, skin development, peptide cross-linking, water homeostasis, and establishment of skin barrier (Fig. 5g). The keratinization pathway was also activated by *FOXC1* depletion (Fig. 5h).

Taken together, these results indicated that dysfunction of FOXC1 disrupted the corneal epithelial identity, and induced the conversion of the corneal epithelium to keratinized epidermis. We identified FOXC1 as a key regulator of the corneal epithelial fate determination and homeostasis.

### FOXC1 maintains corneal epithelial homeostasis via interferon signaling pathways

**Fig. 6:**
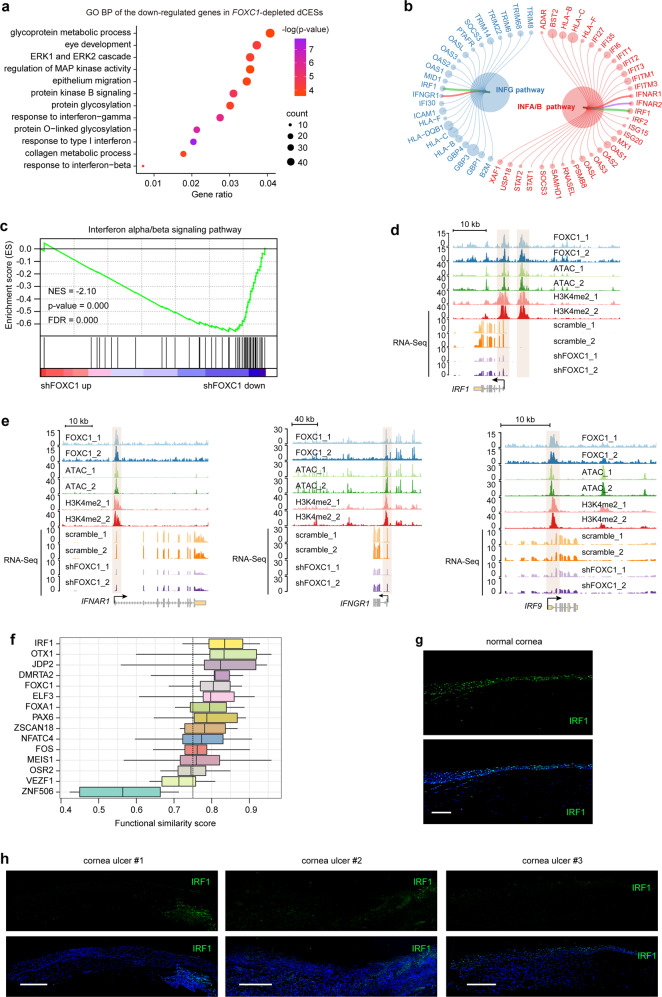
FOXC1 regulates interferon signaling pathways in the corneal epithelium. **a** GO BP analysis of the genes downregulated in *FOXC1*-depleted dCESs. **b** Downregulated interferon alpha/beta/gamma (IFN A/B/G) signaling pathway genes in *FOXC1*-depleted dCESs. Node size represents fold change. **c** GSEA of IFN A/B signaling pathway in the gene expression matrix of scrambled shRNA-treated versus shFOXC1-treated dCESs. **d**, **e** Genome browser tracks for FOXC1, H3K4me2, and ATAC signals in LSCs, and RNA-Seq signals in the dCESs around the *IRF1*, *IFNAR1*, *IFNGR1*, and *IRF9* loci. **f** GSSM of the top 15 (fold change) downregulated TFs in *FOXC1*-depleted dCESs. The cutoff value (0.75) is indicated by the dashed line. These 15 TFs were also highly expressed in the scramble group. **g** Immunofluorescence staining of IRF1 in adult limbus-cornea. Scale bar, 100 μm. **h** Immunofluorescence staining of IRF1 in cornea ulcer tissues. Scale bar, 100 μm

To further explore the role of FOXC1 in corneal epithelial homeostasis, we analyzed the FOXC1-regulated gene network in dCESs. We found that FOXC1 controlled the biological processes of eye development, epithelium migration, and multiple signaling pathways, like regulation of ERK1/ERK2 cascade, regulation of mitogen-activated protein kinase (MAPK) activity, and protein kinase B signaling (Fig. 6a). Importantly, *FOXC1* deficiency disrupted CEC differentiation-associated protein glycosylation, the glycoprotein metabolic process, and collagen metabolic process (Figs. 6a and 3e). Previous study has demonstrated that interferon signaling can inhibit angiogenesis by regulating the expression of vascular endothelial growth factor A (VEGFA) and soluble VEGF receptor 1 in the human cornea.^37^ Remarkably, we found that molecules involving in interferon signaling pathways (*IRF1*, *IFNAR1*, *IFNAR2*, *IFNGR1*, *IFNGR2*, *IRF9*, *STAT1*, and *STAT2*) and genes response to interferon signaling were significantly repressed in *FOXC1*-compromised dCESs (Fig. 6a–c and Supplementary Fig. S6a, b). FOXC1 regulated their expression by directly binding to open promoters or distal enhancers (Fig. 6d, e and Supplementary Fig. S6c).

Moreover, we used GSSM to quantify GO semantic similarity of the top 15 (fold change) downregulated TFs with high expression levels, and showed that interferon-induced IRF1 had the highest functional similarity with others (Fig. 6f), suggesting its hub role. IRF1 also showed a high GO semantic similarity score among the CEC differentiation-associated TFs (Fig. 3h). *IRF1* expression was also inhibited in LSCs when *FOXC1* was knocked down (Supplementary Fig. S3b). The IRF1 motif was enriched in the open chromatin regions in LSCs (Supplementary Fig. S6d). We observed robust expression of IRF1 in the limbus and central corneal epithelium (Fig. 6g). Likewise, loss of IRF1 occurred in the lesion regions of the corneal ulcer tissues (Fig. 6h). These observations indicated that FOXC1 maintains corneal epithelial homeostasis via interferon signaling pathways.

## Discussion

Human corneal epithelium, as a barrier tissue of the eye, suffers from external insults, thus requiring continuous self-renewal and differentiation of resident adult stem cells. LSCs are essential for the maintenance of corneal epithelial homeostasis and visual acuity.^38^ Previous reports demonstrated that FOXC1 is required for ocular anterior segment development in mice and humans, and functional studies on this gene mainly focused on its antiangiogenic role in corneal stroma.^10,11,39,40^ Here, we identified FOXC1 as a core TF that maintains the corneal epithelial identity during adulthood, which suggested a broader role of FOXC1 in the ocular system.

Specifically, several signaling pathways (such as interferon signaling, ERK1/ERK2 cascade, MAPK, and protein kinase B signaling) were affected in *FOXC1*-depleted dCESs, which indicated that these signal pathways may be important for the corneal epithelial identity. In addition, FOXC1 also controlled protein glycosylation and collagen metabolism in dCESs. Previous findings indicated that FOXC1 in neural crest-derived corneal stroma cells maintains corneal transparency and avascularity by regulating proangiogenic matrix metalloproteinases and VEGF signaling.^11^ Thus, FOXC1 appears to govern distinct biological processes in different tissues.

The present study revealed the chromatin binding profile of FOXC1 in LSCs. The function of TFs in the nucleus depends on the active chromatin environment. Thus, we also mapped the chromatin accessibility and H3K4me2 landscapes. As expected, FOXC1 controlled the corneal epithelial gene network by directly binding to promoters or enhancers marked by ATAC and H3K4me2 signals. Notably, motifs of several regulators important to corneal epithelial self-renewal and differentiation were co-enriched at the centers of ATAC and FOXC1 peaks. The proximity of these TFs implied the potential cooperation between them. This observation supported the notion that cooperative binding of cell-type-specific TFs orchestrates lineage identity and homeostasis.^26,41,42^ Collectively, our present work provides novel insights into the FOXC1-mediated regulatory network and epigenetic features required for corneal epithelial homeostasis.

Interestingly, we found that FOXC1 activated gene expression of the whole interferon signaling pathways in dCESs. Interferon signaling robustly represses angiogenesis in human corneal stroma and tumors.^37,43^ Thus, we speculated that FOXC1 could inhibit neo-angiogenesis by regulating interferon signaling in the limbus and corneal epithelium. The anti-angiogenic function of FOXC1 may also be achieved in corneal stromal cells via interferon signaling. Previous report has revealed that the interferon response gene IRF1 is important for the corneal innate immune response to bacterial infection.^37^ Of note, the expression patterns of FOXC1, PAX6, and IRF1 were consistent in corneal epithelial lineage and pathological cornea. Loss of FOXC1, IRF1, and PAX6 was observed in some regions of the corneal ulcer tissues. These findings raised the possibility that disturbance of the FOXC1/interferon signaling axis causes disorder of the corneal innate immune defense, which results in pathological changes in the corneal epithelium. The role and regulatory mechanism of IRF1 in corneal epithelium fate and homeostasis need to be explored in the future.

## Materials and methods

### Clinical materials

Human embryonic cornea was obtained voluntarily from the donor in compliance with the Ethics Committee of The Third Affiliated Hospital of Guangzhou Medical University (2018006). All human pathological tissues were obtained with the approval of the Ethics Committee of Zhongshan Ophthalmic Center of Sun Yat-sen University (2020KYPZ115).

### Culture of primary LSCs

Human limbus tissues isolated from donors were cut into small pieces and digested at 37 °C with 0.2% collagenase IV (Gibco) for 2 h and then with 0.25% trypsin-EDTA (Gibco) for 15 min. LSC expansion was achieved in Matrigel-coated dishes. The components of the LSC medium have been described previously.^8^

### Air-lifting culture protocol

LSCs were plated in transwell culture inserts (Corning), and the medium was added to the upper and lower compartments. The cells were cultured for approximately 3–6 days until they reached confluency, after which the medium was removed from the inserts. LSCs were induced to differentiate for another 5–14 days to form dCESs through contact with the medium in the lower chamber. The medium used for air-lifting culture was the same as that used to culture LSCs.

### Immunofluorescence staining

Immunofluorescence experiments were performed as previously described.^8^ Briefly, after fixation, dehydration, paraffin embedding, and de-paraffinization, the tissue sections were permeated and blocked with 0.3% Triton X-100 and 3% bovine serum albumin for 1 h. Then, the samples were incubated with primary antibodies overnight and with secondary antibodies for 1 h on the following day. Cell nuclei were stained with 4′,6-diamidino-2-phenylindole (DAPI). The following antibodies were used: anti-FOXC1 (Bethyl Laboratories, A303-519A), anti-K19 (Thermo Scientific, MS-1902-P), anti-p63 (BioLegend, 619002), anti-Ki67 (CST, 9129S), anti-Cytokeratin 1 (Invitrogen, MA1-06312), anti-Cytokeratin 10 (Invitrogen, MA1-06319), anti-PAX6 (BioLegend, 901301), anti-Cytokeratin 12 (Abcam,ab124975), anti-Cytokeratin 3 (Abcam, ab68260), and anti-ZNF750 (Sigma, HPA023012).

### RNA interference and quantitative real-time PCR (qPCR) analysis

Two shRNAs targeting *FOXC1* were respectively cloned into the PLKO.1 lentivirus plasmid. LSCs were infected with lentivirus particles encoding shRNAs for 16 h and then selected with 2 μg/mL puromycin (Gibco) for 48 h. The RNeasy Mini Kit (Qiagen) was used to isolate total RNA from cells, and cDNAs were synthesized using the PrimeScript™ RT Master Mix Kit (Takara). qPCR was performed using the iTaq™ Universal SYBR® Green Supermix Kit (Bio–Rad). The following shRNAs were used:

shFOXC1-1: 5′-GAGCTTTCGTCTACGACTGTA-3′

shFOXC1-2: 5′-GTCACAGAGGATCGGCTTGAA-3′

shPAX6-1: 5′-CGTCCATCTTTGCTTGGGAAA-3′

shPAX6-2: 5′-AGTTTGAGAGAACCCATTATC-3′

Scramble: 5′-CAACAAGATGAAGAGCACCAA-3′

### ChIP-Seq

Chromatin was fixed with 1% formaldehyde for 10 min and sheared in a sonification buffer (50 mM HEPES-NaOH, pH 7.5, 300 mM NaCl, 1 mM EDTA, 0.1%, Na-deoxycholate, 1% TritonX-100, and 0.1% SDS). Fragmented DNA was incubated with primary antibodies overnight at 4 °C and then with Protein A/G Dynabeads (1:1, Invitrogen) for 1 h. The beads were collected and washed sequentially with the following solutions: high-salt buffer (50 mM HEPES-NaOH, pH 7.5, 500 mM NaCl, 1 mM EDTA, 0.1%, Na-Deoxycholate, 1% TritonX-100, and 0.1% SDS), low-salt wash buffer (10 mM Tris-HCl, pH 8.0, 250 mM LiCl, 1 mM EDTA, 0.5% IGEPAL CA-630, and 0.5% Na-Deoxycholate), and TE buffer (10 mM Tri-HCl, pH 8.0, and 1 mM EDTA).

Elution of the immunoprecipitated protein/DNA complexes was performed using elution buffer (50 mM Tris-HCl, pH 8.0, 10 mM EDTA, and 1% SDS) at 65 °C for 4 h. Next, the protein/DNA complexes were incubated with proteinase K (Invitrogen) and RNase A (Invitrogen) for 1 h. De-crosslinked DNA was purified using the PCR Purification Kit (Qiagen), and then, the KAPA Hyper Prep Kit (Kapa Biosystems, KK8502) was used to construct DNA libraries for Illumina PE150 sequencing.

### ATAC-Seq

In total, 50,000 cells were digested, collected, and lysed in ice-cold lysis buffer (10 mM Tris-HCl, pH 7.5, 10 mM NaCl, 3 mM MgCl_2_, 0.5% IGEPAL CA-630, and 0.1% Tween-20) for 5 min. Then, Tn5 transposase reactions (Vazyme Biotech, TD501) were performed using the TruePrep DNA Library Prep Kit (Vazyme Biotech). After purification, the transposed DNA fragments were amplified and collected according to manufacturer’s instructions. Library DNA was sequenced with an Illumina HiSeq X10 instrument.

### RNA-Seq data assay

STAR software^44^ was used to align the RNA-Seq reads to the human hg19 reference genome, and the RSEM tool^45^ was used to calculate the TPM values. Differentially expressed genes were identified using the DESeq2 R package,^46^ with a log_2_ fold-change value of ≥1 and a false-discovery rate of <0.05. GO biological process enrichment analysis was performed using the clusterprofiler R package^47^ with a *p*-value cut-off of 0.01 and a *q*-value cut-off of 0.05.

### ChIP-Seq and ATAC-Seq data assays

BWA software^48^ was used to map trimmed reads to the human hg19 reference genome, and the uniquely mapped reads were selected using the Picard MarkDuplicates tools (http://broadinstitute.github.io/picard/). For ChIP-seq data, we used MACS2^49^ to call peaks with the following parameters: -f BAMPE -B –SPMR -q 0.001–call-summits–fix-bimodal–seed 11521 and–extsize 200. For ATAC peak calling, the following parameters were used: -f BAMPE -B –SPMR -q 0.001–call-summits–seed 11521–nomodel–shift -100–extsize 200. The HOMER mergePeaks command was used to obtain overlapping peaks between two biological replicates. Peak distributions were examined with the ChIPseeker package.^50^ Peak annotations were performed using the HOMER’s annotatePeaks.pl program with the default parameters. Peak summits were used to identify motifs using HOMER’s findMotifsGenome.pl program with the parameter: -size 200.

### GSSM analysis

Bioconductor R package GOSemSim was used to compute GO semantic similarity between genes according to the published method.^22^ Functional similarity of genes was defined as the geometric mean of their semantic similarities in molecular function and cellular component of GO analysis.

## Supplementary information

supplementary data

## Data Availability

The data sets that support the findings of this study are available in this paper or the Supplementary Information. Next-generation high-throughput sequencing raw data are available from the corresponding author upon reasonable request.
