# Supplementary material for: Loss of FOXC1 contributes to the corneal epithelial fate switch and pathogenesis
Source: Signal Transduct Target Ther. 2021 Jan 8;6:5. doi: 10.1038/s41392-020-00378-2 (PMC7791103; doi:10.1038/s41392-020-00378-2)
Supplement: Supplementary file 1 — supplementary data [file 41392_2020_378_MOESM1_ESM.docx]

Supplementary Materials for

Loss of FOXC1 contributes to the corneal epithelial fate switch and pathogenesis

Mingsen Li^1^, Liqiong Zhu^1^, Jiafeng Liu^1^, Huaxing Huang^1^, Huizhen Guo^1^, Li Wang^1^, Lingyu Li^1^, Sijie Gu^1^, Jieying Tan^1^, Jing Zhong^1^, Bowen Wang^1^, Zhen Mao^1^, Yong Fan^2^, Chunqiao Liu^1^, Jin Yuan^1^, and Hong Ouyang ^1,3^

Correspondence to: Jin Yuan (yuanjincornea@126.com), Hong Ouyang ([Ouyhong3@mail.sysu.edu.cn](mailto:Ouyhong3@mail.sysu.edu.cn))

**This PDF file includes:**

Figures. S1 to S6.

**Figure. S1.**

**
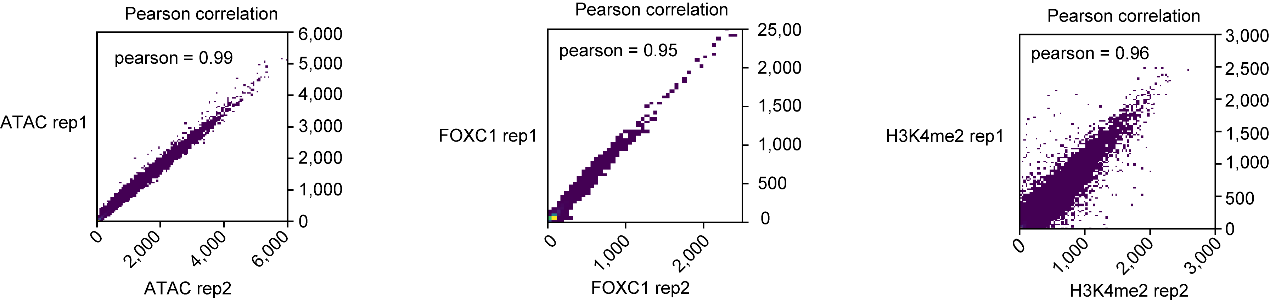
**

**Supplementary Fig.1 High degree of similarity between two biological replicates of ChIP-seq and ATAC-seq data.** Pearson's correlation coefficient of ChIP-seq and ATAC-seq data between two biological replicates in LSCs.

**Figure. S2.**


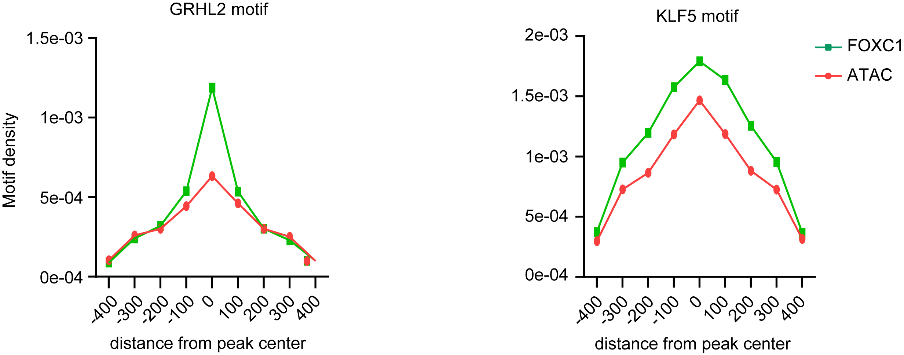


**Supplementary Fig.2 Motif enrichment in the FOXC1 and ATAC binding sites.** Frequencies of GRHL2 and KLF5 motifs around the center of the FOXC1 and ATAC peaks.

**Figure. S3.**


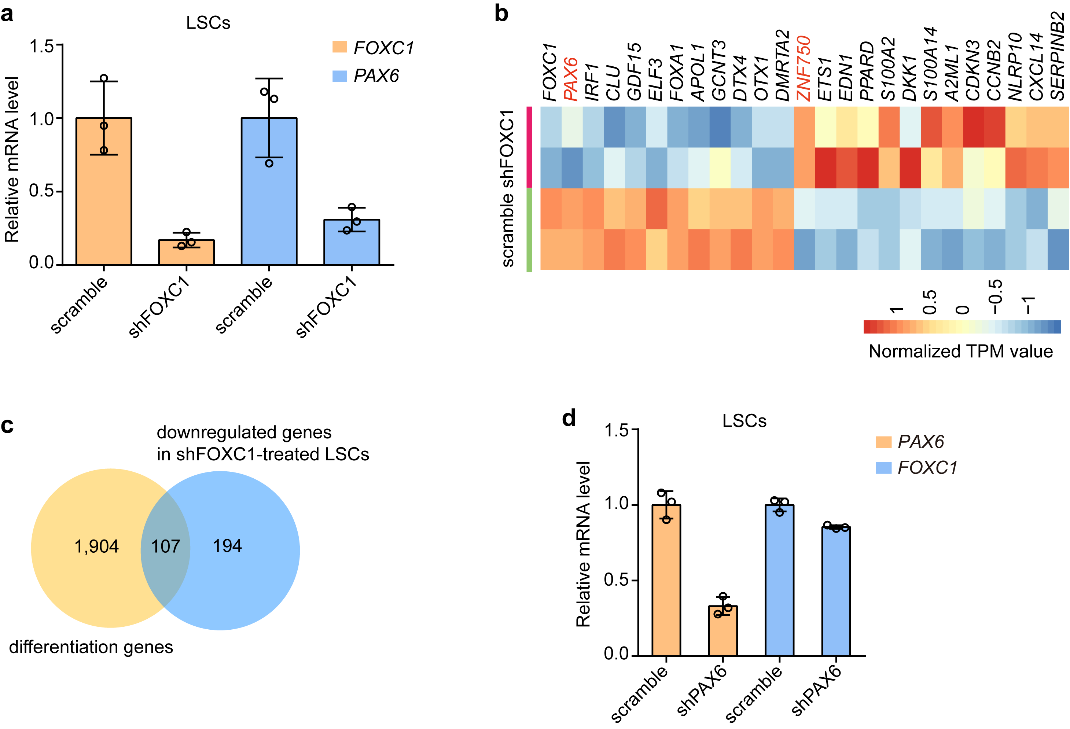


**Supplementary Fig.3 Gene expression change in *FOXC1*-depleted LSCs. a** Quantitative real-time PCR (qPCR) analysis showing the relative expression level of the indicated genes in scrambled shRNA- and shFOXC1-treated LSCs. **b** Heatmap of the selected differentially expressed genes in scrambled shRNA- versus shFOXC1-treated LSCs. **c** Venn diagram showing the overlap between genes preferentially expressed in the dCESs and those that were downregulated after *FOXC1* knockdown in LSCs. **d** qPCR analysis showing the relative expression levels of *FOXC1* and *PAX6* in scrambled shRNA- and shPAX6-treated LSCs.

**Figure. S4.**


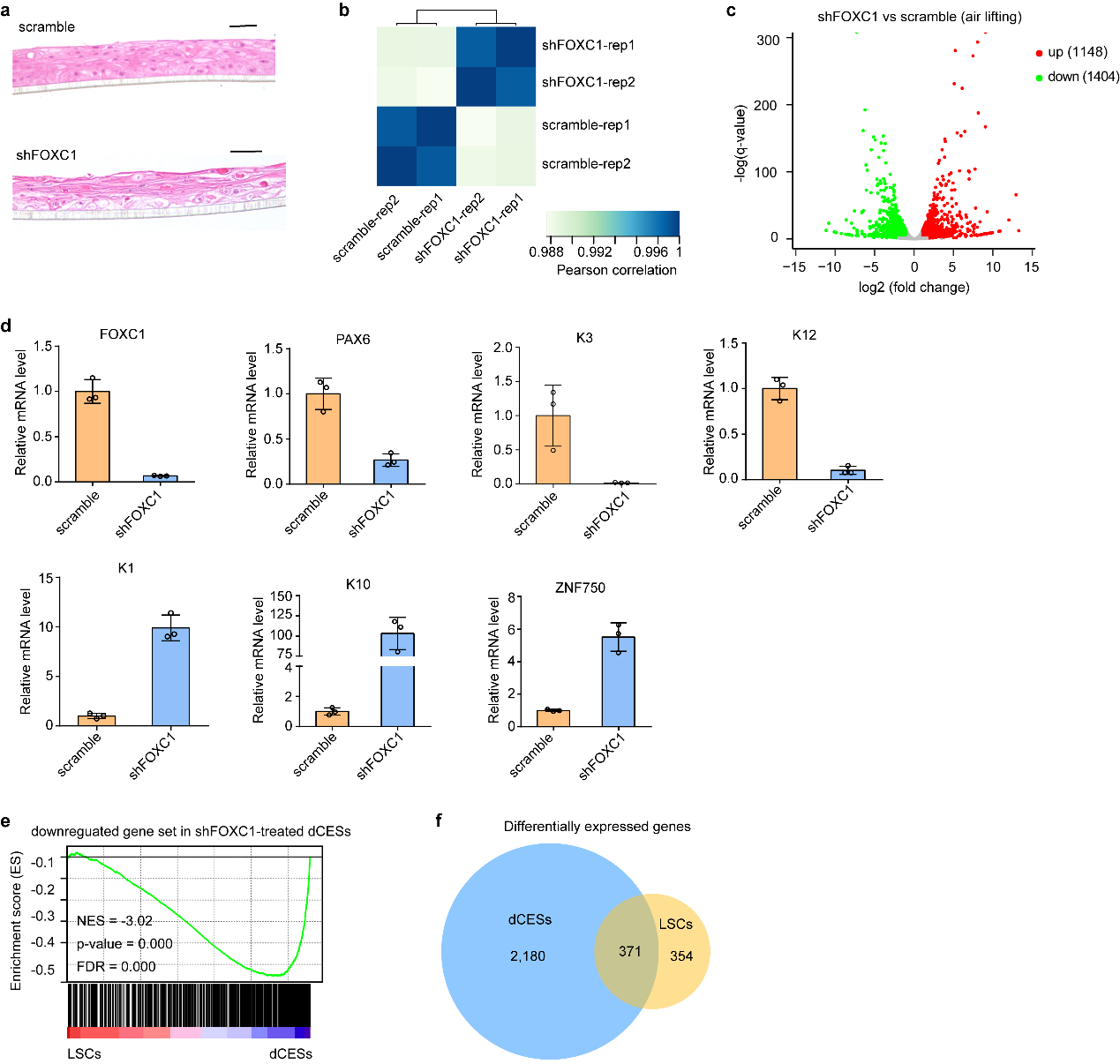


**Supplementary Fig. S4 Gene expression change in *FOXC1-*depleted dCESs. a** Hematoxylin and eosin staining of scrambled shRNA- and shFOXC1-treated dCESs. Scale bar, 50 μm. **b** Hierarchical clustering of RNA-Seq data for scrambled shRNA- and shFOXC1-treated dCESs. **c** Volcano plot showing the differentially expressed genes in scrambled shRNA- versus shFOXC1-treated dCESs. **d** qPCR analysis showing fold changes in the expression levels of the indicated genes in scrambled shRNA- versus shFOXC1-treated dCESs. **e** GSEA of the downregulated gene set induced by *FOXC1* depletion in the gene expression matrix of LSCs versus dCESs. **f** Venn diagram showing the overlap between the differentially expressed gene sets that were induced by *FOXC1* knockdown respectively in LSCs and dCESs.

**Figure. S5.**


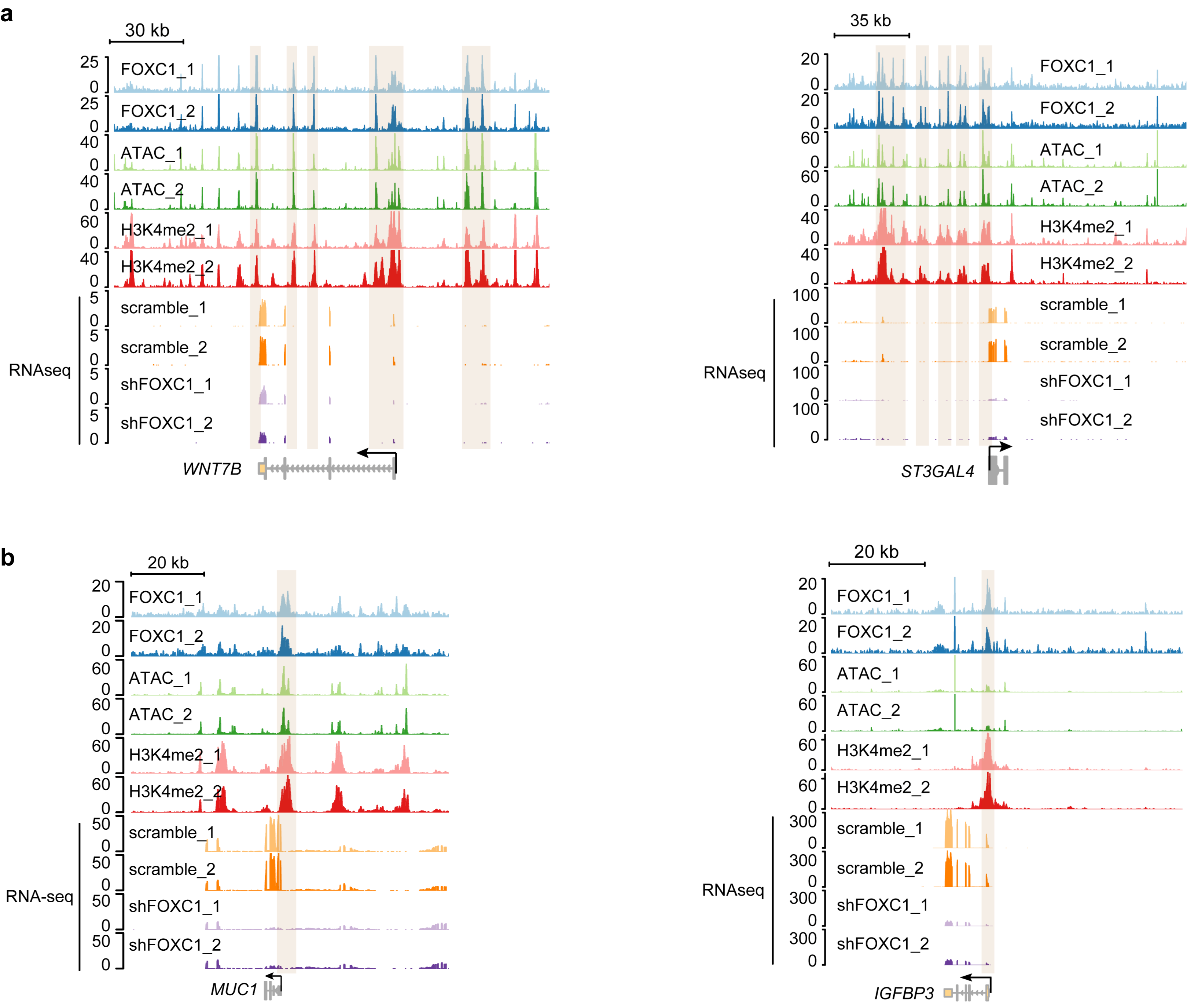


**Supplementary Fig. S5 FOXC1 occupies enhancers or promoters of target genes to control their expression.** Genome browser tracks for FOXC1, H3K4me2, and ATAC signals in LSCs, and RNA-Seq signals in the dCESs around the *WNT7B*, *ST3GAL4*, *MUC1*, and *IGFBP3* loci.

**Figure. S6.**


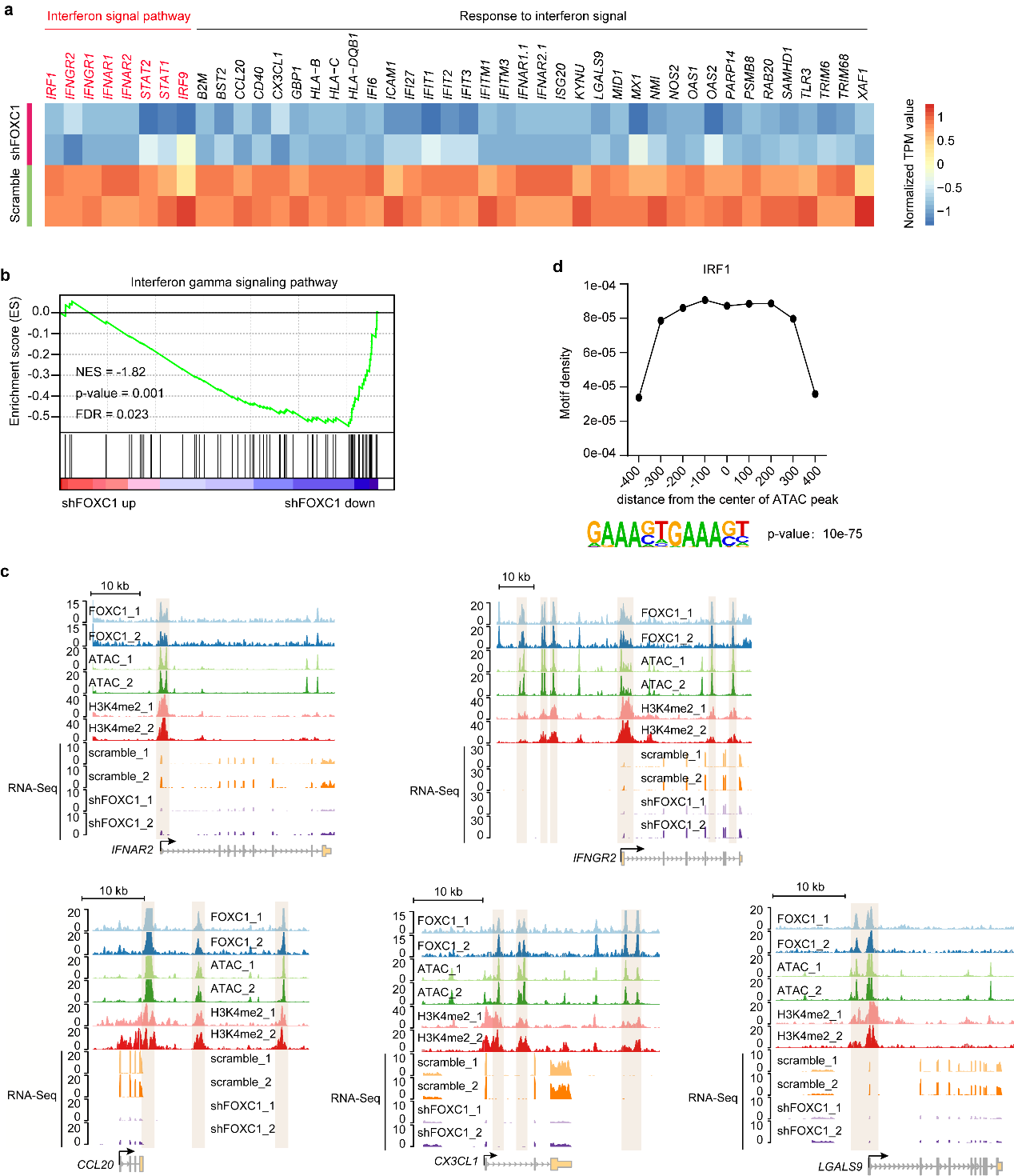


**Supplementary Fig. S6 FOXC1 directly regulates interferon signaling pathway genes. a** Heatmap of the interferon signaling pathway-associated genes in control and shFOXC1-treated dCESs. **b** GSEA of interferon gamma signaling pathway in the gene expression matrix of scrambled shRNA- versus shFOXC1-treated dCESs. **c** Genome browser tracks for FOXC1, H3K4me2, and ATAC signals in LSCs, and RNA-Seq signals in the dCESs around the indicated loci. **d** Motif enrichment of IRF1 in the ATAC peaks.
